# A Composite Photocatalyst Based on Hydrothermally-Synthesized Cu_2_ZnSnS_4_ Powders

**DOI:** 10.3390/ma11010158

**Published:** 2018-01-19

**Authors:** Shih-Jen Lin, Jyh-Ming Ting, Kuo-Chin Hsu, Yaw-Shyan Fu

**Affiliations:** 1 Department of Materials Science and Engineering, National Cheng Kung University, Tainan 701, Taiwan; andy.sjlin@gmail.com; 2 Department of Greenergy, National University of Tainan, Tainan 701, Taiwan; kuochin1225@gmail.com

**Keywords:** Cu_2_ZnSnS_4_ powders, silver nanoparticles, 1T-2H MoS_2_, photodegradation

## Abstract

A novel composite photocatalyst based on Cu_2_ZnSnS_4_ (CZTS) powders was synthesized and investigated for use as a photocatalyst. CZTS powders were first made using a conventional hydrothermal method and were then used to grow silver nanoparticles hybridized onto the CZTS under various conditions through a microwave-assisted hydrothermal process. After the obtained samples were subsequently mixed with 1T-2H MoS_2_, the three synthesized component samples were characterized using X-ray diffractometry (XRD), scanning electron microscopy, transmission electron microscopy (FE-SEM, FE-TEM), UV-visible spectroscopy (UV-Vis), Brunauer-Emmet-Teller (BET), photoluminescence spectroscopy (PL), and X-ray photoelectron spectroscopy (XPS). The resulting samples were also used as photocatalysts for the degradation of methylene blue (MB) under a 300 W halogen lamp simulating sunlight with ~5% UV light. The photodegradation ability was greatly enhanced by the addition of Ag and 1T-2H MoS_2_. Excellent photodegradation of MB was obtained under visible light. The effects of material characteristics on the photodegradation were investigated and discussed.

## 1. Introduction

A photocatalytic technique is considered a promising method for treating organic dyes in wastewater [1,2]. I_2_-II-IV-VI_4_ quaternary chalcogenide semiconductors Cu_2_ZnSnS_4_ (CZTS) are non-toxic, Earth-abundant, and inexpensive, and they have a high absorption coefficient >10^−4^ cm^2^ for potential application as solar-cell absorbers [3,4,5]. However, due to the fact that their optimum band gap is ~1.5 eV, they easily recombine into photogenerated holes and electrons. Thus, it is necessary to reduce the recombination rate. TiO_2_ is a well-known n-type metal oxide photocatalyst in many photocatalytic applications, including hydrogen generation and water splitting in environmental and energy-related fields [1,6,7,8,9]. However, its relatively large band gap of 3.2 eV is only a small fraction (4%) of the solar spectrum. Several studies have investigated ways to achieve better absorption and charge carrier mobility under visible light through the use of a dopant [10,11,12,13]. Koichi Awazu et al. labeled this new phenomenon “plasmonic photocatalysis”, where silver (Ag) nanoparticles (NPs) are coated with a passive material such as SiO_2_ to prevent oxidation and to separate TiO_2_. Their work indicated effective surface plasmon resonance transfer of photogenerated holes on the surface enhanced photocatalytic reaction [14].

Recently, it has been shown that silver orthophosphate (Ag_3_PO_4_) has extremely high photo-oxidative capabilities for O_2_ generation from water splitting under visible light irradiation [15]. GO–Ag_3_PO_4_ nanocomposites have been synthesized as an excellent hybrid photocatalyst [16,17]. They not only exhibit enhanced visible light photocatalytic activity, but also show better stability and durability than their counterparts. Among them, molybdenum disulfide (MoS_2_) exhibits a graphene-like layered structure in which S–Mo–S covalent bonds exist within the plane, and van der Waals forces hold the plane [2,18]. MoS_2_ provides a high surface area, unique electrical conductivity consisting of 1T-2H hybridized phases, and a sheet-like morphology [19]. It has received attention for application in energy storage devices due to its unique nanostructure and electronic properties. 

The photocatalysts of the I_2_-II-IV-VI_4_ quaternary group exhibit better absorption ability under visible light than TiO_2_. Therefore, Xuelian Yu et al. prepared CZTS-Pt and CZTS-Au heterostructured NPs using a synthesis route [20]. The results showed enhanced photocatalytic activity toward photodegradation and hydrogen generation by water splitting. Feng Jiang et al. reported a multilayer of Pt/In_2_S_3_/CdS/CZTS heterostrucures, where the obtained photocathode showed significant enhancement in terms of stability during photoirradiation for PEC water splitting [21]. Hybridized 1T-2H MoS_2_ was then added to provide higher electrical conductivity and the specific surface area necessary to achieve superior catalytic ability [22,23,24]. Importantly, Ag was added in the structure to extend the light absorption of the photocatalyst to the visible light region, which was due to the surface plasmon resonance of Ag NPs, which enabled the photocatalyst to absorb light in the visible spectrum. Owing to these outstanding characteristics, we report a novel design of three-component Ag/hybridized CZTS/1T-2H MoS_2_ heterojunction photocatalysts using a facile hydrothermal method. Since their appropriate band gap structures and stability enhance properties that include light harvesting, charge separation, and transfer photoactivity, it is essential to develop this method on a photocatalytic mechanism.

## 2. Results and Discussion

### 2.1. Crystal Structure

**Figure 1:**
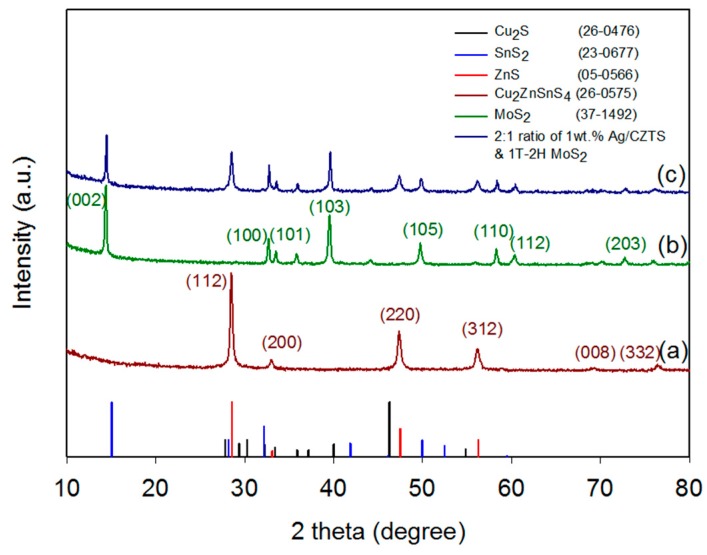
XRD pattern of (**a**) CZTS powders using the conventional hydrothermal process (CHT) at 180 °C for 72 h; (**b**) MoS_2_ powder using the CHT process at 200 °C for 24 h; (**c**) 2:1 ratio of 1 wt% Ag/CZTS and 1T-2H MoS_2_.

The XRD patterns of the Kesterite (KS) CZTS, 1T-2H MoS_2_, and 1 wt% Ag-CZTS/1T-2H MoS_2_ are shown in Figure 1. Figure 1a shows that the major CZTS diffraction peaks observed at 28.47°, 32.99°, 47.35°, 56.14°, 68.99°, and 76.42° can be indexed to the crystal planes of (112), (200), (220), (312), (008), and (332) of the KS structure (JCPDS no. 26-0575), respectively. The formation mechanisms of the CZTS powder are summarized as Cu_2_S + SnS_2_ + ZnS → Cu_2_ZnSnS_4_. It is necessary for the excess thiourea concentration to be a homogeneous solution in order to effectively induce a self-assembly reaction through the conventional hydrothermal (CHT) method [4]. No secondary phases of Cu_2_S, SnS_2_ or ZnS were detected [25]. Figure 1b shows that the CHT synthesized MoS_2_ microflower sample obtained the major diffraction peaks at 14.42°, 32.69°, 33.49°, 39.52°, 49.76°, and 58.3°, which correspond to the (002), (100), (101), (103), (105), and (110) crystalline planes of MoS_2_ (JCPDS no. 37-1492), respectively. In addition, the calculated interlayer spacing (d-002) using the Bragg equation was 0.66 nm, with the reported well-controlled expansion of the interlayer d-spacing in the hydrothermal/solvothermal approaches ranging from 0.62 to 1.1 nm [26]. In Figure 1c for the 1 wt% Ag/CZTS and 2:1 ratio of 1T-2H MoS_2_ composite, no peaks were observed for Ag probably due to the very small amount of Ag added onto the CZTS. The XRD pattern corresponding to both the CZTS and MoS_2_ peaks can be observed.

### 2.2. Morphology

**Figure 2:**
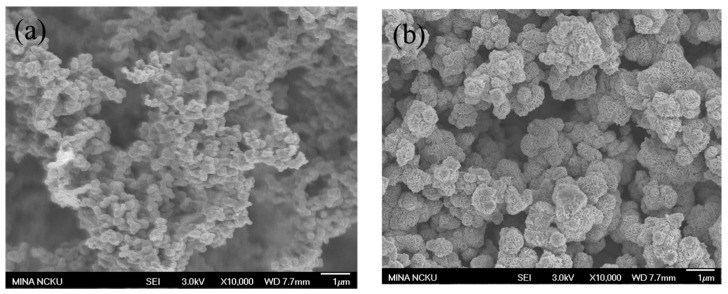

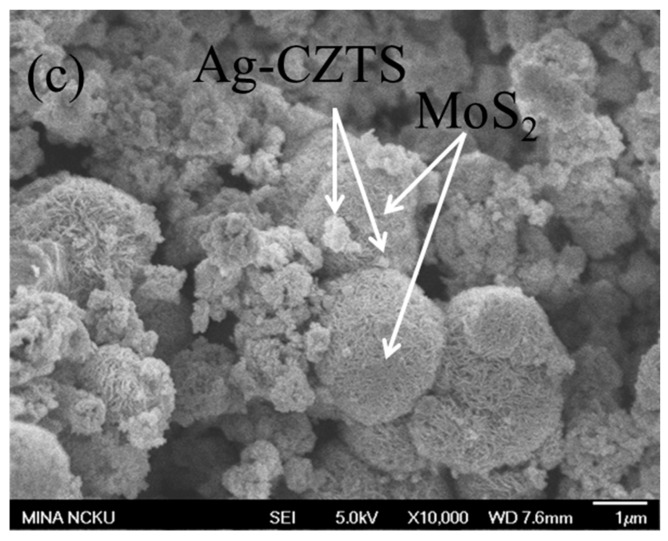
Scanning electron microscopy (SEM) images of the prepared CZTS powders with the (**a**) 1 wt% Ag doped on CZTS powder; (**b**) 1T-2H MoS_2_ microflower; and (**c**) the 2:1 ratio of 1 wt% Ag/CZTS and 1T-2H MoS_2_.

The morphology of Ag/CZTS/1T-2H MoS_2_ NPs based on the SEM results is shown in Figure 2. Figure 2a shows the SEM images of 1 wt% Ag hybridized KS CZTS NPs. Shapes with a minor amount of irregular particles were obtained, but the addition of Ag on the CZTS was not observed on the SEM images probably because the Ag particles were too small to be detected. Figure 2b shows the images of CHT-synthesized MoS_2_ microflowers obtained as the petals, which consist of several MoS_2_ nanosheets. On the other hand, Figure 2c shows that the morphologies of the as-prepared 2:1 ratio of 1 wt% Ag/CZTS and 1T-2H MoS_2_ are well grown on the surface of the MoS_2_ powder, thereby successfully forming the Ag/CZTS/1T-2H MoS_2_ heterostructure.

**Figure 3:**
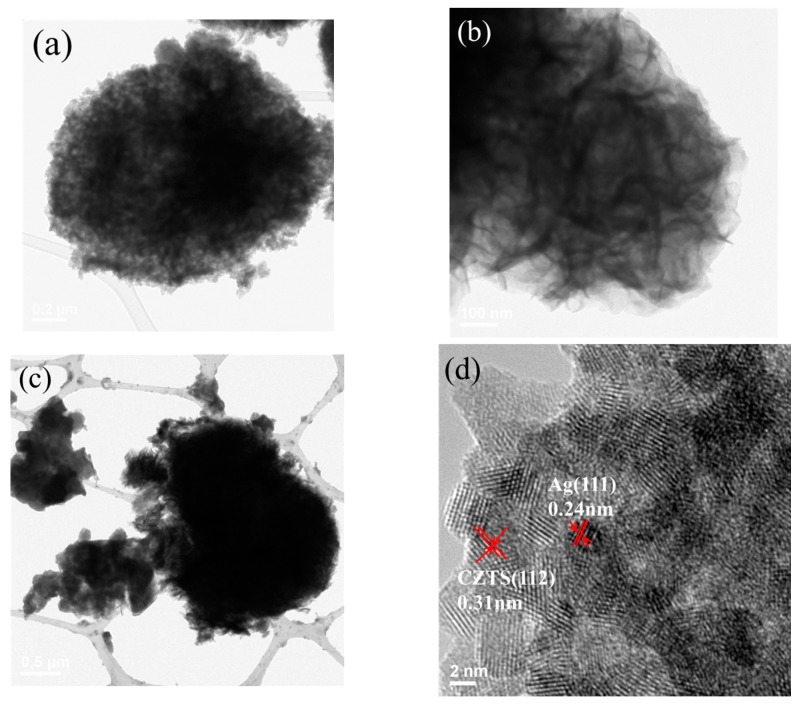

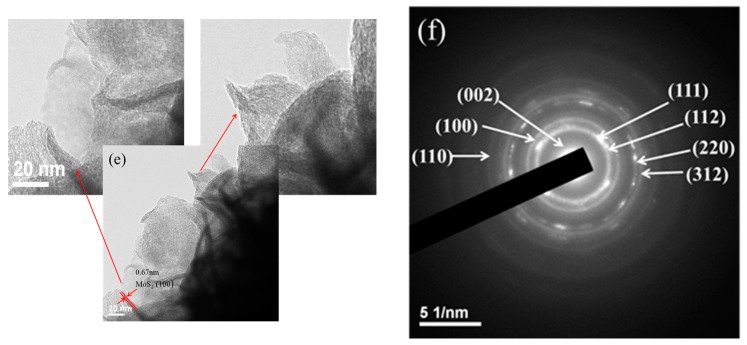
TEM images (**a**) 1 wt% Ag doped on CZTS powders; (**b**) 1T-2H MoS_2_ microflower; (**c**) 2:1 ratio of 1 wt% Ag/CZTS and 1T-2H MoS_2_; HRTEM images of (**d**) 1 wt% Ag/CZTS; (**e**) 1T-2H MoS_2_ microflower; and (**f**) SAED micrographs of 2:1 ratio of 1 wt% Ag/CZTS and 1T-2H MoS_2_.

Figure 3a shows the TEM images of Ag/CZTS powders, where the Ag NPs with a darker contrast were hybridized on the CZTS powder. Figure 3d shows the high resolution TEM images wherein the interplannar lattice spacing of 0.31 nm corresponds to the (112) planes [5]. Meanwhile, the additional Ag corresponds to the face-centered cubic (FCC) Ag (111) [27], with a spacing of 0.24 nm. Figure 3b,e show that the microflower morphologies of MoS_2_ consist of a considerable number of single and few layer structures. The MoS_2_ lattice fringe spacing was also calculated, for which the corresponding d-spacing was 0.67 nm [22]. Figure 3c,f show the more aggregated nanoparticles of the 1 wt% Ag/CZTS and 2:1 ratio of 1T-2H MoS_2_ and the SAED image of the 1 wt% Ag/CZTS and 2:1 ratio of 1T-2H MoS_2_, which is consistent of with tetragonal KS CZTS, as it shows the major phases (112), (220), and (312), indicating that the hybridizing of the Ag NPs can be attributed to the diffraction from the (111) plane of FCC. The corresponding SAED results for the 1T-2H MoS_2_ microflower match the (002), (100), and (110) planes [23,24].

### 2.3. Surface Areas

**Figure 4:**
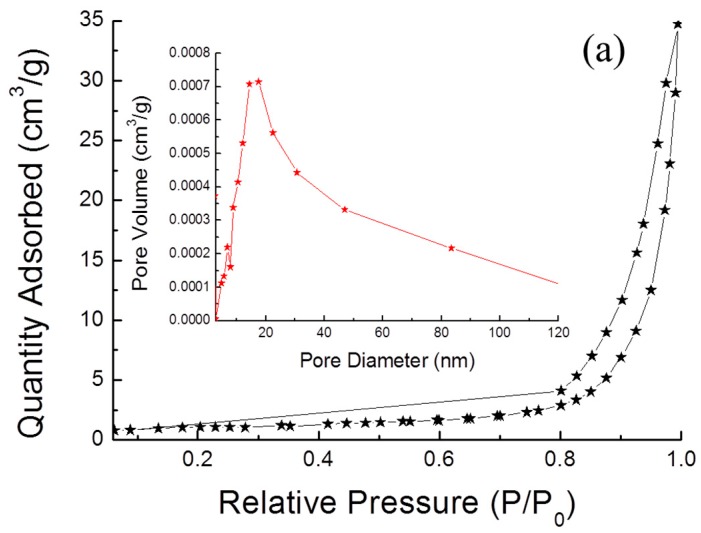

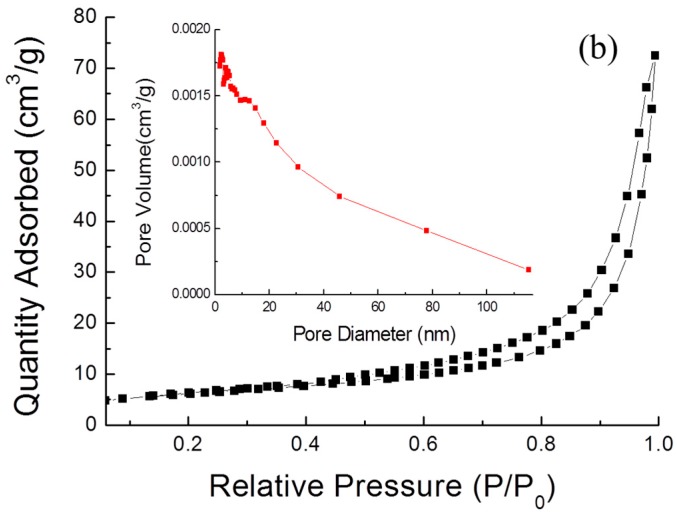
N_2_ absorption/desorption isotherms of (**a**) CZTS powders, and (**b**) 1T-2H MoS_2_ microflower powders; insets are BJH pore size distribution plots, respectively.

Figure 4 shows the Brunauer–Emmett–Teller (BET) analysis using N_2_ adsorption-desorption isotherms and the corresponding Barrett–Joyner–Halenda (BJH) pore size distributions curves (inset). The surface areas of the CZTS powders and 1T-2H MoS_2_ are measured as 3.38 and 21.39 m^2^ g^−1^, respectively. The insets are the BJH pore volume are 0.045 and 0.096 cm^3^ g^−1^, and the pore size distribution plot, for which the adsorption average pore diameters are 31.74 and 18.02 nm, respectively. It can be seen that there are differences in the morphology results in different specific surface areas (SSAs), which indicates that the MoS_2_ has a higher SSA than is the case for the CZTS powders due to the fact that the microflower shape provided a higher surface area. However, the pore size of the 1T-2H MoS_2_ is smaller than that of the CZTS powders. This may be because the alkaline solution of diethylenediamine (DETA) acted as an etchant and increased the excess thiourea, which is an intermolecular force and polarity molecule during the synthesis of the CZTS powders, resulting in the formation of more pits on the surface [28,29]. Therefore, the larger SSA and pore diameter in the Ag/CZTS/MoS_2_ contributed to active enhancement of photo-catalytic efficiency [24].

### 2.4. Optical Absorption

**Figure 5:**
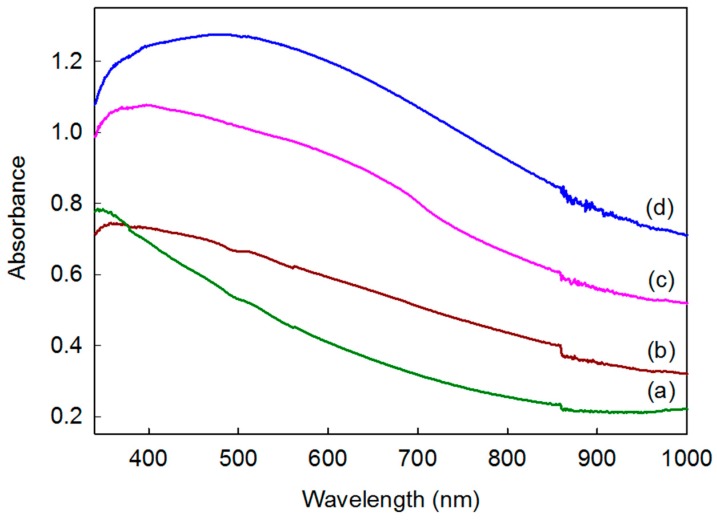
UV-VIS absorption spectra of (**a**) CZTS; (**b**) 1 wt% Ag/CZTS; (**c**) 10:1 ratio of 1 wt% Ag/CZTS and 1T-2H MoS_2_; and (**d**) 2:1 ratio of 1 wt% Ag/CZTS and 1T-2H MoS_2_.

The UV-VIS absorption spectrum results for the tetragonal KS CZTS powders, 1 wt% Ag/CZTS, 1 wt% Ag/CZTS, and 10:1 and 2:1 ratios of 1T-2H MoS_2_ are shown in Figure 5. It can be seen that doping Ag enhanced the light absorption, and this fact can be ascribed to the charge transfer process from the valence band to the conduction band by board surface plasmon resonance (SPR) absorption in the visible region [30]. However, further increases in the 10 wt% and 50 wt% 1T-2H MoS_2_ microflower powder resulted in broad absorption due to a higher SSA. It is apparent that a high surface area enhances the visible light absorption. This is likely due to the strong monotonic absorption in the visible light region of the hybridized 1T-2H MoS_2_. Among the samples, the 2:1 ratio of 1 wt% Ag/CZTS and 1T-2H MoS_2_ exhibited the best enhancement. 

### 2.5. PL Analysis

**Figure 6:**
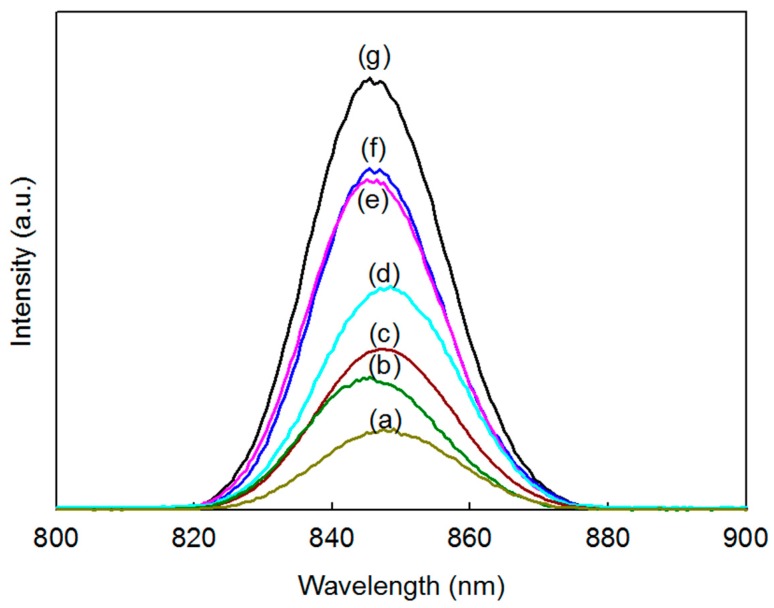
PL spectra of (**a**) 2:1 ratio of 1 wt% Ag/CZTS and 1T-2H MoS_2_; (**b**) 10:1 ratio of 1 wt% Ag/CZTS and 1T-2H MoS_2_; (**c**) 1 wt% Ag/CZTS; (**d**) 2 wt% Ag/CZTS; (**e**) CZTS; (**f**) 4 wt% Ag/CZTS; and (**g**) 8 wt% Ag/CZTS.

A PL analysis was then performed to determine the effect of Ag on the recombination rate of the photocatalyst. Figure 6 shows that the PL intensity was greatly increased when a 4 and 8 wt% of Ag was added to the CZTS photocatalyst, and a further decrease was observed when the amount was decreased to 2 and 1 wt%. This suggested that the electrons promoted in the conduction band of CZTS jump to the surface plasmon state of Ag, thus reducing the recombination rate of the structure. However, excessive amounts of electrons were generated in the structure, which caused the Ag nanoparticles to serve as a recombination site in the system [30]. On the other hand, 1 wt% Ag/CZTS hybridized a higher SSA of MoS_2_ that absorbed the visible light and efficiently transferred the photogenerated charge carriers. The CZTS nanoparticles generated excited electrons and holes from its valence band to its conduction band after which the electrons generated in the CZTS conduction band jumped to the surface plasmon state of the Ag nanoparticles to be either directly transferred to MoS_2_ or shuttled across the Ag to the MoS_2_. Finally, the presence of hybridized Ag and 1T-2H MoS_2_ microflower powders might indicate a decrease in the recombination rate necessary for photocatalysis.

### 2.6. XPS Study

**Figure 7:**
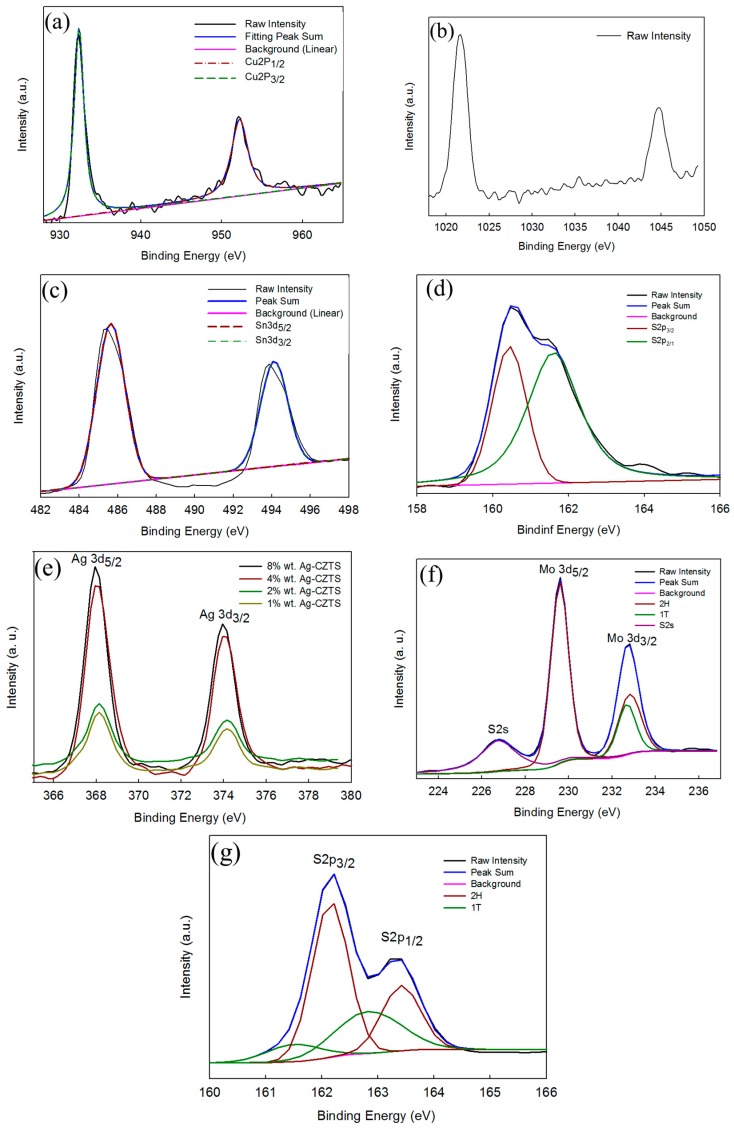
High-resolution XPS characterization of as-synthesized (**a**) Cu 2p; (**b**) Zn 2p; (**c**) Sn 3d; (**d**) S 2p; (**e**) Ag NPs; (**f**) Mo 3d; and (**g**) S 2p.

A calibration was made for each sample with respect to 284.6 eV of carbon C 1s. The XPS analysis carried out on the four constituent elements, copper 2p, zinc 2p, tin 3d, and sulfur 2p. (Figure 7a), showed the binding energy of the Cu 2p_3/2_ and Cu 2p_1/2_ peaks at 931.4 and 952.2 eV, respectively, with a peak splitting at 19.8 eV, suggesting Cu (I). Figure 7b showed the Zn 2p_3/2_ and Zn 2p_1/2_ peaks at 1021.8 and 1044.8 eV, respectively, with a peak splitting at 23 eV, corresponding to Zn (II). Figure 7c shows the Sn 3d_5/2_ and Sn 3d_3/2_ peaks at 485.8 and 494.2 eV, respectively, with peak splitting at 8.4 eV, which is in a good agreement with Sn (IV). Figure 7d shows the S 2p_3/2_, S 2p_1/2_ peaks at 161.1, 162.3 eV, respectively, from S (II) [5]. The Ag 3d XPS spectra of Ag/CZTS samples obtained from different weight percentages of AgNO_3_ precursor: 1, 2, 4, and 8 wt% are shown in Figure 7e. The high resolution spectra of the Ag 3d core level for the samples showed two peaks, which were located around 368.2 eV and 374.3 eV. These peaks proved that AgNO_3_ was successfully reduced to metallic Ag through the microwave-assisted hydrothermal process in water as a solvent [31]. Further confirmation of the existence of the hybridized 1T-2H phase of MoS_2_ is provided in Figure 7f, where a typical MoS_2_ microflower shows Mo 3d_5/2_ and Mo 3d_3/2_ peaks at 229.7 eV and 232.7 eV for the 2H phase, respectively, and an S 2s peak at 226.6 eV in the high resolution Mo 3d spectrum. Furthermore, a minor peak at 235.7 eV appears to the Mo^6+^ 3d_5/2_. However, the samples shift toward lower energy peaks at 228.7 and 231.9 eV for the 1T phase, which shifts toward a lower binding energy with a difference of ~0.8 eV with respect to the peaks in the 1T-2H phase [18,19]. Similarly, Figure 7g shows the S 2p XPS spectra of the MoS_2_ samples indicating that two additional signatures at 161.7 eV and 162.8 eV appear beside the two S 2p_3/2_ and S 2p_1/2_ 2H MoS_2_ peaks at 162.6 eV and 163.7 eV, respectively. These two peaks also shift to the lower binding energy side and are associated with the 1T phase [19].

### 2.7. Photocatalytic Activity

**Figure 8:**
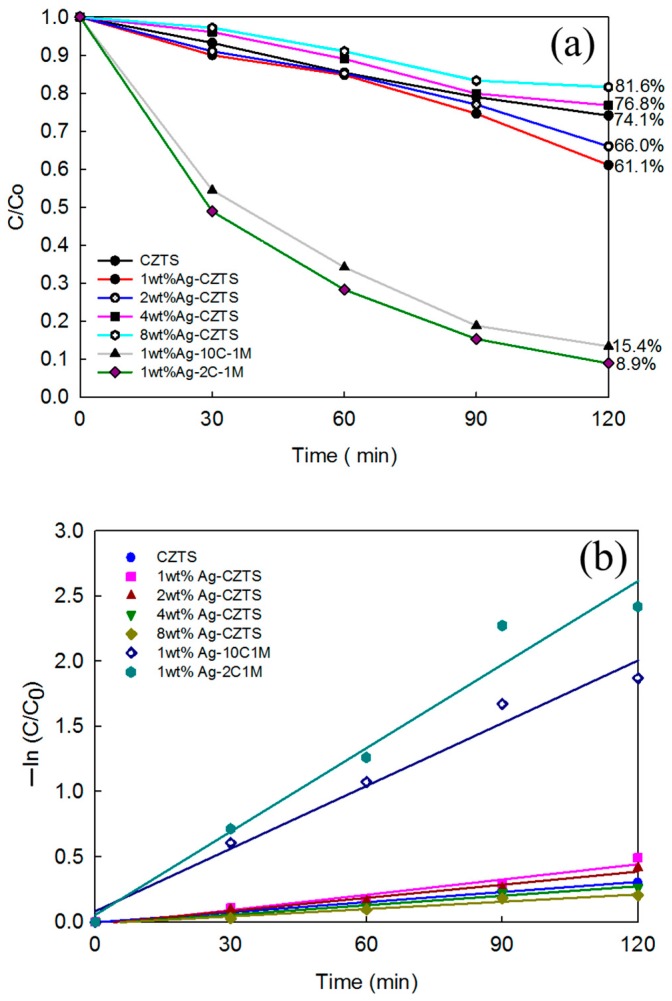
Photocatalytic activity under solar irradiation through the hybridized various weight percentages of AgNO_3_ precursor: 1, 2, 4, and 8 wt% Ag/CZTS and the ratios of 10:1 and 2:1 of 1 wt% Ag/CZTS and 1T-2H MoS_2_; (**a**) the degradation of MB; and (**b**) the photocatalytic degradation kinetics evolution.

**Figure 9:**
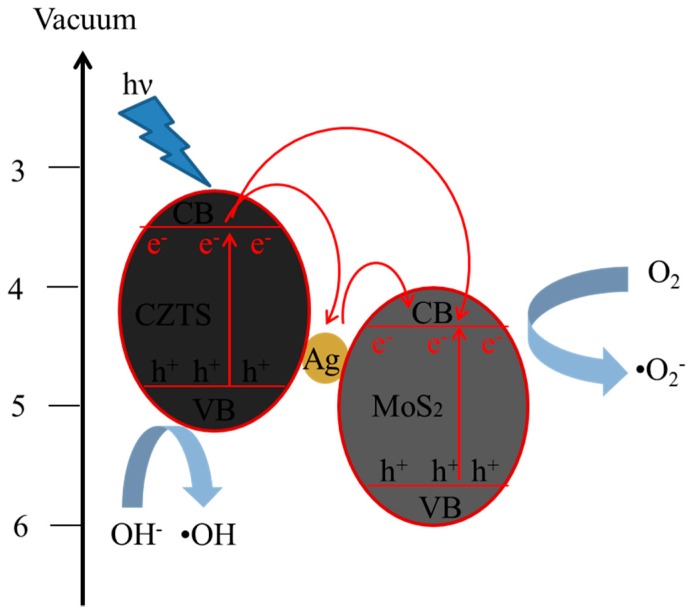
Schematic diagram for proposed photodegradation mechanism of methylene blue solution in the presence of the Ag/CZTS hybridized 1T-2H MoS_2_ photocatalyst.

The photocatalytic performance of all of the Ag/CZTS and hybridized 1T-2H MoS_2_ photocatalysts were measured and compared with the photocatalytic performance. The curves of C/C_0_, where C_0_ is the initial concentration of MB, and C is the reaction concentration of MB at time t are shown in Figure 8a. It can be seen that the hybridized Ag decreased as the Ag content increased. In the case of the addition of 1T-2H MoS_2_, regardless of whether the ratio was lower (10 wt%) or was 1 wt%, Ag doped on the CZTS greatly enhanced the photocatalytic performance. Figure 8b shows the first order linear transform, which was determined by plotting –ln (C/Co) versus irradiation time. The corresponding k values for the CZTS, 1, 2, 4, 8 wt% Ag, the 1 wt% Ag/CZTS, and the 10:1 and 2:1 ratios of the 1T-2H MoS_2_ microflower powders samples were estimated to be 0.0025, 0.0039, 0.0033, 0.0024, 0.0019, 0.016, and 0.0213 min^−1^, respectively. The photodegradation rate constant of the three component Ag/CZTS/1T-2H MoS_2_ sample was faster than that for the CZTS and Ag/CZTS samples. A schematic diagram of the proposed mechanism is provided in Figure 9. The results were attributed to the combined effects of enhanced visible light absorption by Ag and the efficient charge separation due to the MoS_2_ in the photocatalyst system. Due to the surface plasmon resonance, electrons below the Fermi level of the Ag nanoparticles excited to their plasmonic state. Then, the electrons generated in the CZTS conduction band jumped to the surface plasmon state of the Ag nanoparticles, thus reducing the recombination rate. The electrons generated in and transferred to the Ag nanoparticles reacted to O_2_ gas to form superoxide radicals (^•^O_2_^−^). These active radicals reacted with the methylene blue solution to degrade it into H_2_O and CO_2_. On the other hand, the holes generated in the system reacted with the adsorbed H_2_O to form hydroxyl radicals (^•^OH). These active radicals also reacted with the methylene blue solution to degrade it into H_2_O and CO_2_.

## 3. Materials and Characterization

### 3.1. Materials

All chemicals were used as received without further purification. Analytical grade materials included copper (II) chloride anhydrous (CuCl_2_, Choneye pure chemicals Co., Ltd., Taipei City, Taiwan, 98%), zinc chloride (ZnCl_2_, Merck, Kenilworth, NJ, USA, 98%), stannous chloride dehydrate (SnCl_2_∙2H_2_O, Shimakyu, Osaka, Japan, 98%), thiourea (NH_2_CSNH_2_, Riedel-de Haën, Mexico City, Mexico, 99%), diethylenediamine (C_4_H_13_N_3_, Panerac quimica sa, Barcelona, Spain, 98%), silver nitrate (AgNO_3_, Aencore, Surrey Hills, Australia, 99.8%), sodium molybdate dihydrate (NaMoO_4_∙2H_2_O, J.T. Baker, Center Valley, PA, USA, 99.5%), and oxalic acid (Riedel-de Haën, 99.5%), and the deionized water (DI water) used was from EMD Millipore Corporation.

### 3.2. CZTS Convental Hydrothermal Process

Deionized water was used as the solvent. In a typical aqueous synthesis, 30 mmol of thiourea was dissolved in 20 mL of deionized water in a 50 mL Teflon-lined autoclave and stirred for 15 min. 2.5 mmol of ZnCl_2_ and SnCl_2_·2H_2_O were dissolved in 5 mL of deionized water, and 5 mmol of CuCl_2_ (anhydrous) was used in 10 mL of deionized water. All of the precursors were stirred for 10 min individually and were then poured into the 50 mL Teflon-lined stainless-steel autoclave, with the addition 0.543 mL diethylenediamine (DETA) as the chelating agent. The autoclave was sealed and maintained at 180 °C for 72 h and then allowed to cool down to room temperature naturally. After hydrothermal synthesis, the solution was washed and centrifuged with dimethylformamide and deionized water three times to remove the byproducts and DETA. Black particles were then obtained by drying in an oven at 80 °C for 8 h.

### 3.3. Ag/CZTS Microwave-Assisted Hydrothermal Process

The obtained CZTS powers were charged 0.75 g then various amounts of AgNO_3_ solution were added (1 wt%, 2 wt%, 4 wt%, and 8 wt% in 20 mL deionized water). The solution was then loaded into a Teflon-lined autoclave at 200 °C for 1 h in a microwave-assisted system for the hydrothermal process. Finally, the products were washed three times with ethanol and DI water, centrifuged, and dried overnight in a vacuum oven at 60 °C to obtain the 1, 2, 4, and 8 wt% Ag/CZTS powders.

### 3.4. MoS_2_ Conventional Hydrothermal Process

MoS_2_ synthesized from 5 mmol NaMoO_4_·2H_2_O and 5 mmol oxalic acid were dissolved in 20 mL DI water and then stirred for 20 min. Then, 20 mmol thiourea was added and dissolved in 10 mL DI water separately. The solutions were then loaded into a 50 mL Teflon-lined autoclave for the hydrothermal process at 200 °C for 24 h. The MoS_2_ powders were then collected and washed three times with ethanol and DI water. They were then dried at 60 °C overnight in a vacuum oven.

### 3.5. Ag/CZTS/MoS_2_ Hybridized Process

Finally, three components of the Ag/CZTS/MoS_2_ composite were mixed in 20 mL of ethanol with the varying weight ratios of 1 wt% Ag/CZTS and MoS_2_ powder 10:1 and 2:1 for 1 h. The products were washed three times with DI water and ethanol and then dried at 60 °C overnight in a vacuum oven.

### 3.6. Characterization

The crystalline structure of the powders was examined using X-ray diffraction (XRD Bruker D8 advance, Billerica, MA, USA). The morphology was determined using field emission scanning electron microscopy (FESEM, JEOL JSM-6701F, Tokyo, Japan). The microstructure was investigated using transmission electron microscopy (TEM, JEOL JEM-2100F, Tokyo, Japan). X-ray photoelectron spectroscopy (XPS, ULVAC-PHI, Inc. PHI 5000 VersaProbe, Chigasaki-shi, Japan) spectra were obtained using a 1486.6 eV Al anode X-ray source. XPS peak 4.1 software was used for the peak fitting, where the resulting peak components were pure Gaussian. The surface area was measured according to Brunauer-Emmet-Teller (BET, Micromeritics Instrument Corp. Tristar II, Norcross, GA, USA). The optical properties of particle suspensions were determined using a UV-VIS absorption spectrum (PerkinElmer Lambda 7500, Waltham, MA, USA). The PL spectra were obtained using a LS-55 fluorescence spectrometer (PerkinElmer, Waltham, MA, USA).

### 3.7. Photocatalytic Behavior

Methylene blue (MB), which is widely utilized in organic dye textile industries, is typically used in photocatalytic dye degradation studies. All the samples were carried out using 20 mg of the photocatalysts in 60 mL of 20 ppm methylene blue solution. The solution was stirred in the dark for 30 min to achieve the adsorption-desorption equilibrium. Furthermore, stirring was continued under a 300 W halogen lamp intended to simulate sunlight with ~5% UV light. Then, ~4 mL of the charged solution was collected every 30 min and centrifuged for 15 min to remove the photocatalyst completely. The concentration of MB solution was measured with the absorbance at 664 nm with the UV–VIS spectrophotometer (PerkinElmer Lambda 7500, Waltham, MA, USA).

## 4. Conclusions

In this paper, we discuss the use of a conventional hydrothermal method for the synthesis of Cu_2_ZnSnS_4_ powders well indexed into a high-purity KS phase. Furthermore, hybridized Ag/CZTS was successfully synthesized through a microwave-assisted hydrothermal process using water as the solvent for such processes as ultrasound and microwave irradiation to facilitate chemical reactions. It was found that the photocatalytic performance of the CZTS powders was greatly improved by the addition 1 and 2 wt% of Ag NPs deposited on the surface of the CZTS acting as the electron traps of the matrix. This prevented the recombination of electron-hole pairs and improved the charge transfer processes by hybridizing the 1T-2H MoS_2_ in the structure, thus increasing the light harvesting in photocatalysis due to the broad light absorption range. This technique was attributed to the strong enhancement of visible light absorption and efficient charge separation, leading to reduced recombination. A maximum of 91.1% MB degradation occurred in under 2 h, where the photocatalyst containing a 2:1 ratio of 1 wt% Ag/CZTS and 1T-2H MoS_2_ exhibited the highest photodegradation.
